# Association of Ambient Air Pollution with Depressive and Anxiety Symptoms in Older Adults: Results from the NSHAP Study

**DOI:** 10.1289/EHP494

**Published:** 2016-08-12

**Authors:** Vivian C. Pun, Justin Manjourides, Helen Suh

**Affiliations:** Department of Health Sciences, Northeastern University, Boston, Massachusetts, USA

## Abstract

**Background:**

Ambient fine particulate matter (PM_2.5_) is among the most prevalent sources of environmentally induced inflammation and oxidative stress, both of which are implicated in the pathogenesis of most mental disorders. Evidence, however, concerning the impact of PM_2.5_ on mental health is just emerging.

**Objective:**

We examined the association between PM_2.5_ and current level of depressive and anxiety symptoms using a nationally representative probability sample (*n* = 4,008) of older, community-dwelling individuals living across the United States (the National Social Life, Health and Aging project).

**Methods:**

Mental health was evaluated using validated, standardized questionnaires and clinically relevant cases were identified using well-established cutoffs; daily PM_2.5_ estimates were obtained using spatiotemporal models. We used generalized linear mixed models, adjusting for potential confounders, and explored effect modification.

**Results:**

An increase in PM_2.5_ was significantly associated with anxiety symptoms, with the largest increase for 180-days moving average (OR = 1.61; 95% CI: 1.35, 1.92) after adjusting for socioeconomic measures (SES); PM_2.5_ was positively associated with depressive symptoms, and significantly for 30-day moving average (OR = 1.16; 95% CI: 1.05, 1.29) upon SES adjustment. The observed associations were enhanced among individuals who had low SES and history of comorbidity. When considering mental health as chronic conditions, PM_2.5_ was significantly associated with incident depressive symptoms for all exposure windows examined, but with incident anxiety symptoms only for shorter exposure windows, which may be due to a drop in power resulting from the decreased between-subject variability in chronic PM_2.5_ exposure.

**Conclusion:**

PM_2.5_ was associated with depressive and anxiety symptoms, with associations the strongest among individuals with lower SES or among those with certain health-related characteristics.

**Citation:**

Pun VC, Manjourides J, Suh H. 2017. Association of ambient air pollution with depressive and anxiety symptoms in older adults: results from the NSHAP study. Environ Health Perspect 125:342–348; http://dx.doi.org/10.1289/EHP494

## Introduction

Mental health disorders accounted for over 140 million disability-adjusted life years worldwide in 2010 (Whiteford et al. 2013) and is the third most costly non-fatal condition in the United States, totaling $60 billion annually (Soni 2011). Adult mental disorder rates are substantial, with 18% experiencing anxiety disorder and 9.8% major depressive, dysthymic, and bipolar disorder in the past year (NIH/NIMH 2015). One of the hypothesized biological pathways is that mental disorders occur through oxidative stress and neuro-inflammation pathways (Ng et al. 2008; Vogelzangs et al. 2013). Compared to other organs, the brain is vulnerable to oxidative stress damage because of its high-energy use, low-endogenous scavenger levels, high-metabolic demands, and high-cellular lipid and protein content (Halliwell 2006; Mattson 2001). It is also susceptible to secondary and self-perpetuating damage from oxidative cellular injury via activated neuro-inflammatory responses or other pathways (Halliwell 2006; Ng et al. 2008). While genetic profiles, brain damage, substance abuse, socioeconomic status, and life situations have been important risk factors of mental disorders, recent evidence has indicated a role of physical environmental factors in the pathogenesis of mental disorders.

Airborne particulate matter (PM) pollution is a major source of environmentally induced inflammation and oxidative stress (Block and Calderón-Garcidueñas 2009). Ambient PM exposure has been consistently linked to adverse cardiovascular and respiratory effects, with oxidative stress and systemic inflammation considered the primary pathways through which air pollution damages health (Brook et al. 2004, 2010). While epidemiologic studies in the 1980s suggested associations between air pollution and mood (Bullinger 1989), depressive symptoms (Jacobs et al. 1984), and psychiatric emergencies (Rotton and Frey 1984), only recently have studies examined the possible effects of PM on mental illness and stress, with conflicting results (Lim et al. 2012; Marques and Lima 2011; Mehta et al. 2015; Power et al. 2015; Szyszkowicz 2007; Szyszkowicz et al. 2009; Wang et al. 2014). Toxicological studies, however, have shown neuropathological effects (e.g., increased levels of pro-inflammatory cytokines, degenerated dopaminergic neurons) and neurobehavioral responses (e.g., depression-like behaviors) upon PM exposure (Calderón-Garcidueñas et al. 2003; Campbell et al. 2005; Davis et al. 2013; Fonken et al. 2011; Veronesi et al. 2005). In this study, we used data from the National Social Life, Health and Aging Project (NSHAP) to examine the association of exposure to PM with aerodynamic diameter of ≤ 2.5 μm (PM_2.5_) with current depressive and anxiety symptom severity.

## Methods

### Participants

NSHAP is a longitudinal, nationally representative study of community-dwelling individuals (57–85 years) without known cognitive impairment living across the United States, with oversampling of African-Americans, Hispanics, men, and individuals between 75–84 years old (Shega et al. 2014; Waite et al. 2014a, 2014b). Numerous social, psychological, functional, and physiological health measures were collected for each participant in two data collection waves. Wave 1 was conducted from July 2005 to March 2006, with in-home interviews, biospecimen collection, and respondent completed questionnaires performed for 3,005 individuals. The same data were obtained in Wave 2 (August 2010 to May 2011) for 3,377 participants, including 2,261 Wave 1 respondents, 161 Wave 1 eligible, but non-interviewed respondents, and 955 spouses or cohabitating romantic partners (see Figure S1). Individuals from Wave 1 who did not participate in Wave 2 included those who were either deceased, moved away, or whose health (e.g., stroke) was too poor to participate in Wave 2. Participants and nonrespondents did not differ with regard to air pollution levels and cognitive scores. The overall weighted response rate was 75.5% and 76.9% for Waves 1 and 2, respectively (O’Muircheartaigh et al. 2009; Smith et al. 2009). The protocol was approved by the Institutional Review Boards of Northeastern University, the University of Chicago, and NORC at the University of Chicago. All participants provided written informed consent.

### Mental Health Measures

Current level of depressive symptomatology was assessed using an 11-item form of the Center for Epidemiological Studies–Depression (CESD-11) Scale questionnaire (Kohout et al. 1993). The CESD-11 is a shorter version of the well-validated 20-item CESD (CESD-20) and is a self-reported screening tool that has been shown to capture the same dimensions as CESD-20 with similar precision. Participants were asked to indicate their response to 11 statements (see Table S1). Each statement asked participants to rate the frequency of their feelings during the previous week as rarely or none of the time (0), some of the time (1), occasionally (2), and most of the time (3), corresponding to a 4-point Likert scale. Positively phrased statements were reverse coded before summation (range: 0 to 33), with higher summed scores indicating more severe depressive symptoms. The Cronbach’s alpha for internal consistency was 0.80 for the entire NSHAP sample. A score of ≥ 9 on the CESD-11 was used to identify individuals with moderate-to-severe depressive symptoms based on previous studies (Kohout et al. 1993; Torres 2012).

The Hospital Anxiety and Depression Scale (HADS) has been used successfully as a self-rating instrument to measure current state of anxiety, with well-established reliability and validity in population-based studies (Mykletun et al. 2001). NSHAP participants were asked to complete a 7-item anxiety subscale of HADS (HADS-A) to indicate the frequency of feelings of anxious mood, thoughts, and restlessness over the past week on a 4-point Likert scale (see Table S1) One positively phrased statement was reverse coded. Individual statement scores were then summed to obtain the total HADS-A score (range: 0 to 21), with higher scores indicating increasing levels of anxiety. The Cronbach’s alpha of the HADS-A was 0.76. A HADS-A cutoff score of 8 gives the optimal sensitivity and specificity (approximately 0.80) to categorize individuals as having an anxiety disorder or not (Bjelland et al. 2002). Thus, we defined participants with a cutoff score of ≥ 8 as cases with moderate-to-severe anxiety symptoms.

### Exposure Assessment

Daily PM_2.5_ estimates on a 6-km grid covering the conterminous United States were obtained from a set of five spatio-temporal generalized additive mixed models (GAMMs) of daily PM_2.5_ mass levels in the conterminous United States, fit separately to 1999–2001, 2002–2004, 2005–2007, 2008–2009, and 2010–2011. These models were based on the spatiotemporal GAMM of monthly PM_2.5_ mass from 1999–2007 documented in Yanosky et al. (2014). PM_2.5_ data were obtained primarily from the U.S. Environmental Protection Agency (EPA) Air Quality System database and Interagency Monitoring of Protected Visual Environments (IMPROVE) network (IMPROVE 2013; U.S. EPA 2016). The model included three meteorological covariates (i.e., wind speed, temperature, and total precipitation) that influence pollutant dispersion as well as several geospatial covariates (i.e., smoothed county population density from the 2000 U.S. Census, point-source PM_2.5_ emissions density within 7.5 km, proportion of urban land use within 1 km, elevation, and annual average PM_2.5_ for 2002 from the U.S. EPA’s Community Multiscale Air Quality model). Finally, the daily PM_2.5_ model includes traffic-related PM levels, represented as the output of a Gaussian line-source dispersion modeling approach. The line-source model uses ADMS-Roads software (version 4.0; Cambridge Environmental Research Consultants Ltd.) and associated spatially smoothed traffic intensity and daily meteorological inputs to describe small-scale spatial gradients in primary PM concentrations near roadways. The daily PM_2.5_ model has undergone validation during development using cross-validation techniques (see Yanosky et al. 2014), and had a cross-validation *R* ^2^ of 0.76. NSHAP participants were matched to the grid (*n* = 894 in total) closest to their residential addresses. Two participants were excluded from the study as their residential addresses were outside the conterminous United States. The mean distance between each grid centroid–residential address pair was 2.23 km, with a range of 0.05–4.21 km.

### Statistical Analysis

Given the longitudinal study design and multiple participants per household, we used the generalized linear mixed models PROC GLIMMIX procedure (version 9.3; SAS Institute Inc.) to study the association of PM_2.5_ and each mental health condition, modeled as binary outcome based on a CESD-11 score ≥ 9 and HADS-A ≥ 8 for moderate-to-severe depressive and anxiety symptoms, respectively, and to account for random effects of repeated measurements for participants and households. We fit penalized spline models to evaluate deviations from linearity, with the linear model preferable for each outcome based on Akaike information criterion. We examined associations for PM_2.5_ exposure windows averaged from previous 7 days, to up to 4 years prior to the interview date of NSHAP participants to study the impact of semi-acute and chronic PM_2.5_ exposure for mental disorders, respectively.

In the basic models, we adjusted for age, sex, race, year, season and day of week of questionnaire completion, region of residence (West, Midwest, South, Northeast), and whether participants lived within a metropolitan statistical area (MSA). Multivariable models were also constructed to control for confounding by socioeconomic measures (SES) as assessed using individual-specific education attainment and family income, and census-level median household income and percent of population with income below poverty level. To further evaluate potential confounding, additional wave-specific covariates were selected *a priori* based on their previous associations with mental illness or air pollution: individual-specific obesity status [i.e., body mass index (BMI) ≥ 30)], current smoking status, physical activity, alcohol consumption (drinks per day), UCLA Loneliness scale (range: 0–9), current use of antidepressant medication, and history of diabetes, hypertension, stroke, heart failure, emphysema, chronic obstructive pulmonary disease (COPD) or asthma (see Table S2). Two covariates (i.e., BMI and family income) had 10% and 29% missing data, respectively; their missing values were imputed by simple mean substitution. Missing data of other covariates (< 5%) were not imputed. Both base and SES-adjusted analyses were restricted to a subset of data for which values for all covariates were not missing [i.e., 6,199 nonmissing out of 6,382 total observations (97.1%) for covariates]. Additional covariates were added individually in separate basic models to avoid multicollinearity and reduce potential bias on the estimates if covariates were not shown to be confounders (Xing and Xing 2010). Since certain covariates (e.g., sex) could be possible effect modifiers, their modification of PM_2.5_-mental health findings was examined through interaction terms, using the PROC GLIMMIX procedure, which provides added options to compute customized odds ratios and the corresponding confidence intervals (CIs) automatically for each level of the interaction term.

We conducted several sensitivity analyses. First, we considered mental health measures as continuous rather than binary measures. Second, we restricted the longitudinal analysis to individuals who participated in both waves, to those living in MSAs only, those who did not move between waves or did not currently take antidepressant medication, respectively. We also reanalyzed the models using multiple imputation technique. Third, we constructed the model using PM_2.5_ concentrations measured at the nearest U.S. EPA ambient monitors within 60 km of the residential address. Lastly, we considered our depression and anxiety outcomes to be chronic relapsing disorders, by restricting our analyses to Wave 2 participants who did not have moderate-to-severe depressive (CESD-11 < 9) or anxiety (HADS-A < 8) symptoms in Wave 1. In doing so, we acknowledge that if mental disorders are chronic conditions, PM_2.5_ exposures for Wave 2 could not be associated with mental disorders that occurred at Wave 1 or earlier. If that is the case, inclusion of individuals reporting mental disorders in Wave 1 in longitudinal analyses would bias the effect estimates towards the null. Since information on the history of mental illness was not available in the study, we conducted logistic regression analysis examining the association between PM_2.5_ exposure and incident moderate-to-severe depressive and anxiety symptoms in Wave 2. Results are expressed as the odds ratio (OR) per 5 μg/m^3^ increment in PM_2.5_ exposure; all effect estimates and their corresponding confidence intervals were obtained through the ODDSRATIO (DIFF = ALL) option in the GLIMMIX procedure.

## Results

A total of 4,008 community-dwelling participants were available for analysis. Overall, participants were on average 69 and 72 years old in Wave 1 and 2 respectively, and nearly half were men (Table 1). Most participants were white, exercised ≥ 1 time per week, and had a high school education or greater. Approximately three-fifths of the participants reported a history of high blood pressure or hypertension; one-fifth diabetes, one-sixth emphysema, COPD, or asthma; and 10% or less stroke or heart failure, respectively. Participants reported slightly higher current use of antidepressant medications and lower UCLA Loneliness score in Wave 2 than in Wave 1. The prevalence of current moderate-to-severe depressive symptoms decreased from 24% in Wave 1 to 21% in Wave 2, while that of moderate-to-severe anxiety symptoms increased in Wave 2 (14%) compared with Wave 1 (21%). Four (< 1%) and 744 participants (12%) did not complete the depression or anxiety assessments, respectively, with missingness not related to air pollution exposures. Intra-wave correlation for CESD-11 score was 0.55, and that for HADS-A score was 0.37. The mean annual concentration (± SD) of PM_2.5_ was 11.1 (± 3.0) μg/m^3^ and 8.8 (± 2.2) μg/m^3^ in Wave 1 and 2, respectively (Table 1; see also Table S3). Refer to Table S4 for descriptive characteristics stratified by data collection wave and pollution category.

**Table 1:** Characteristics of NSHAP study participants by wave.

Characteristic	Study population	
	Wave 1 (July 2005–March 2006)	Wave 2 (August 2010–May 2011)
No. of participants	3,005	3,377
Age (year, mean ± SD)	69.3 ± 7.8	72.4 ± 8.1
Male (%)	48.4	45.5
Race (%)
White	70.5	71.5
Black	17.0	15.4
Hispanic nonblack	10.2	10.9
Other	2.3	2.3
BMI (kg/m^2^, mean ± SD)	29.1 ± 6.1	29.3 ± 6.1
Obesity (% ≥ 30 BMI)	35.1	36.7
Alcohol consumption (drinks/day, mean ± SD)	1.1 ± 1.6	0.94 ± 1.4
Current smoking (%)	14.8	13.3
Physical activity (%)
3 or more times per week	61.4	40.9
1–2 times per week	15.4	15.5
1–3 times per month	6.3	8.7
Less than 1 time per month	6.4	9.3
Never	10.4	25.6
Socioeconomic status
Individual level
Education attainment (%)
College degree or greater	21.9	24.5
High school or vocational school	54.9	56.4
Less than high school	23.3	19.1
Family income ($ in thousands, mean ± SD)	51.3 ± 64.4	59.3 ± 74.2
% ≤ $35,000	37.7	31.0
Census level^*a*^
Median household income ($ in thousands, mean ± SD)	52.7 ± 25.1	56.4 ± 27.3
Population with income below poverty level (%)	15.3	14.4
Loneliness score (mean ± SD)	4.0 ± 1.4	3.1 ± 2.3
Diabetes (%)	21.4	23.7
Hypertension (%)	57.3	61.6
Stroke (%)	8.9	9.4
Heart failure (%)	9.5	5.0
Emphysema, COPD or asthma (%)	17.2	15.4
Antidepressant use (%)	12.5	15.2
CESD-11 score (mean ± SD)	5.6 ± 5.2	5.1 ± 4.9
Number (%) ≥ 9	730 (24.3)	703 (20.8)
HADS-A score (mean ± SD)	3.6 ± 3.5	4.7 ± 3.7
Number (%) ≥ 8	378 (13.5)	605 (21.3)
PM_2.5_ annual concentration (μg/m^3^, mean ± SD)	11.1 ± 3.0	8.8 ± 2.3
Note: 2,261 participants were in both Wave 1 and Wave 2; 744 participants were in wave 1 only; 1,114 were in wave 2 only. There was a total of 6,382 observations. BMI, body mass index; CESD, Center for Epidemiological Studies–Depression; COPD, chronic obstructive pulmonary disease; HADS-A, Hospital Anxiety and Depression Scale–anxiety subscale; SD, standard deviation. ^***a***^Estimated for census tract of residence using data from the U.S. Census Bureau (2000).		

The associations of ambient PM_2.5_ in the previous 7, 30, 180, and 365 days and 4 years prior with each measure of mental health are presented in Table 2. In basic models, a 5 μg/m^3^ increase in PM_2.5_ was significantly and positively associated with moderate-to-severe anxiety symptoms for all exposure windows, with the largest increase in odds for 180-days PM_2.5_ exposure (OR = 1.55; 95% CI: 1.31, 1.85). On the other hand, exposure to PM_2.5_ averaged over the previous 7 days and 30 days was significantly associated with 1.09 (95% CI: 1.01, 1.17) and 1.20 times (95% CI: 1.08, 1.33) the odds of moderate-to-severe depressive symptoms, respectively. Elevations in odds, though statistically insignificant, were also seen for longer moving averages. Analysis of an extended range of exposure windows shows that the effect estimates of depressive and anxiety symptoms increase gradually and are the largest at 60-days and 180-days PM_2.5_ exposure, respectively (see Figure S2). Pattern of associations from multivariable models, which further adjusted for SES, were generally consistent to those from basic models (Table 2). Findings were similar in sensitivity analyses *a*) considering mental health measures as linear continuous variables, *b*) controlling for additional covariates, *c*) using an alternative imputation technique, *d*) using different PM_2.5_ exposure measures from nearby ambient monitors, and *e*) restricting to individuals who participated in both waves, lived in MSAs, did not move between waves, or did not currently take antidepressant medication (see Tables S5–S9).

**Table 2:** ORs (95% CIs) for mental illness per 5 μg/m^3^ increment in PM_2.5_ levels over various moving averages.

PM_2.5_ moving averages	Depression: CESD-11 ≥ 9 vs. < 9		Anxiety: HADS-A ≥ 8 vs. < 8	
	Basic^*a*^	Multivariable adjusted^*b*^	Basic^*a*^	Multivariable adjusted^*b*^
7-days	1.09 (1.01, 1.17)*	1.08 (1.00, 1.16)**	1.14 (1.05, 1.24)*	1.14 (1.05, 1.24)*
30-days	1.20 (1.08, 1.33)*	1.16 (1.05, 1.29)*	1.34 (1.19, 1.50)*	1.31 (1.20, 1.51)*
180-days	1.10 (0.94, 1.28)	1.04 (0.89, 1.22)	1.55 (1.31, 1.85)*	1.61 (1.35, 1.92)*
365-days	1.10 (0.93, 1.31)	1.06 (0.89, 1.27)	1.33 (1.10, 1.61)*	1.39 (1.15, 1.69)*
4-years	1.17 (1.00, 1.38)**	1.14 (0.97, 1.34)	1.29 (1.08, 1.54)*	1.34 (1.12, 1.61)*
Note: CESD, Center for Epidemiological Studies–Depression; HADS-A, Hospital Anxiety and Depression Scale–anxiety subscale. ^***a***^Basic models adjusted for age, sex, race/ethnicity, year, season, day of week, region, and residence within a metropolitan statistical area (MSA). ^***b***^Multivariable models adjusted for age, sex, race/ethnicity, year, season, day of week, region, residence within an MSA, education attainment and family income of the participants, and median household income, percentage of population below poverty level in the census tract of residence. *p < 0.05. **p < 0.10.				

Table 3 (see also Table S10) shows evidence of effect modification for the relationship between mental illness and average 30-day PM_2.5_ level, the exposure window that shows generally significant associations. Individuals who had less than a high school education were at significantly higher odds of PM_2.5_-associated moderate-to-severe anxiety symptoms (*p*-interact < 0.001), and suggestive higher odds of moderate-to-severe depressive symptoms. The association of PM_2.5_ and depressive symptoms was also greater for individuals with low census-level SES (i.e., high percentage of population with income below poverty level) or for those with a history of stroke or respiratory illnesses. Participants who had a history of stroke or heart failure also showed increased odds of moderate-to-severe anxiety symptoms associated with PM_2.5_, compared to those who had no such history.

**Table 3:** Effect modification analysis of the association of mental illness with 5 μg/m^3^ increment in PM_2.5_ levels over preceding 30-days moving average in multivariable models with interaction terms for the potential modifier.

Effect modifier	Depression: CESD-11 ≥ 9 vs. < 9		Anxiety: HADS-A ≥ 8 vs. < 8	
	OR (95% CI)	*p*_interact_	OR (95% CI)	*p*_interact_
Sex
Male	1.11 (0.97, 1.27)		1.36 (1.17, 1.58)
Female	1.12 (0.98, 1.27)	0.905	1.39 (1.21, 1.59)	0.793
BMI
< 30	1.09 (0.97, 1.23)		1.40 (1.23, 1.60)
≥ 30	1.14 (0.99, 1.32)	0.579	1.34 (1.13, 1.57)	0.592
Smoking
No	1.10 (0.98, 1.23)		1.37 (1.21, 1.79)
Yes	1.20 (0.98, 1.46)	0.396	1.42 (1.12, 1.55)	0.771
Socioeconomic status
Individual level
Education
College degree or greater	1.12 (0.93, 1.36)		1.20 (0.98, 1.47)
High school or vocational school	1.02 (0.90, 1.16)	0.347	1.28 (1.11, 1.47)	0.583
Less than high school	1.37 (1.15, 1.64)	0.105	1.97 (1.60, 2.41)	< 0.001
Family income
>$35,000	1.15 (1.02, 1.30)		1.39 (1.21, 1.59)
≤$35,000	1.05 (0.91, 1.21)	0.220	1.34 (1.14, 1.58)	0.703
Census-level^*a*^
Median household income
High	1.01 (0.82, 1.24)		1.45 (1.17, 1.81)
Median	1.09 (0.96, 1.24)	0.513	1.43 (1.24, 1.65)	0.895
Low	1.23 (1.04, 1.45)	0.117	1.20 (0.99, 1.45)	0.152
% population with income below poverty level
Low	0.86 (0.70, 1.04)		1.27 (1.03, 1.56)
Median	1.18 (1.04, 1.34)	0.003	1.45 (1.26, 1.66)	0.229
High	1.18 (1.00, 1.40)	0.007	1.28 (1.05, 1.55)	0.938
Diabetes
No	1.10 (0.98, 1.23)		1.33 (1.17, 1.51)
Yes	1.13 (0.95, 1.34)	0.794	1.58 (1.29, 1.93)	0.086
Hypertension
No	1.09 (0.95, 1.25)		1.36 (1.18, 1.56)	
Yes	1.13 (1.00, 1.28)	0.625	1.39 (1.20, 1.62)	0.752
Stroke
No	1.07 (0.96, 1.19)		1.33 (1.18, 1.50)
Yes	1.55 (1.22, 1.98)	0.002	1.84 (1.40, 2.41)	0.018
Heart failure
No	1.14 (1.02, 1.27)		1.32 (1.17, 1.49)
Yes	0.90 (0.69, 1.17)	0.088	1.97 (1.47, 2.63)	0.007
Emphysema, COPD or asthma
No	1.07 (0.95, 1.20)		1.34 (1.18, 1.52)
Yes	1.28 (1.07, 1.53)	0.048	1.48 (1.22, 1.79)	0.305
Note: Multivariable models adjusted for age, sex, race/ethnicity, year, season, day of week, region, residence within a metropolitan statistical area (MSA), education attainment and family income of the participants, and median household income, percentage of population below poverty level in the census tract of residence. CESD, Center for Epidemiological Studies–Depression; COPD, chronic obstructive pulmonary disease; HADS-A, Hospital Anxiety and Depression Scale–anxiety subscale. ^***a***^Estimated for census tract of residence using data from the U.S. Census Bureau (2000).				

When anxiety and depression were considered as chronic relapsing disorders using logistic regression (Table 4), increments of PM_2.5_ levels in all exposure windows were positively and statistically significantly associated with incident moderate-to-severe depressive symptoms in Wave 2, corresponding to 1.35–1.68 times the odds in multivariable models. Increase in PM_2.5_ levels averaged over the previous 7-days was also significantly associated with incident moderate-to-severe anxiety symptoms (OR = 1.36; 95% CI: 1.09, 1.68). The increase and statistical significance in odds of incident moderate-to-severe anxiety symptoms gradually reduced with longer PM_2.5_ exposure windows.

**Table 4:** Logistic regression analysis [ORs (95% CIs)] of the association between mental disorders and 5 μg/m^3^ increment in PM_2.5_ levels over various moving averages—restricting to WAVE 2 participants who did not have moderate-to-severe depressive (CESD-11 < 9) or anxiety (HADS-A < 8) symptoms in Wave 1.

PM_2.5_ moving averages	No moderate-to-severe depressive symptoms in Wave 1 (1,724). Depression: CESD-11 ≥ 9 vs. < 9		No moderate-to-severe anxiety symptoms in Wave 1 (1,551). Anxiety: HADS-A ≥ 8 vs. < 8	
	Basic^*a*^	Multivariable adjusted^*b*^	Basic^*a*^	Multivariable adjusted^*b*^
7-days	1.37 (1.10, 1.70)*	1.35 (1.08, 1.68)*	1.35 (1.09, 1.68)*	1.36 (1.09, 1.68)*
30-days	1.52 (1.14, 2.03)*	1.54 (1.15, 2.05)*	1.24 (0.93, 1.65)	1.22 (0.92, 1.63)
180-days	1.44 (1.04, 2.00)*	1.48 (1.06, 2.06)*	1.10 (0.80, 1.51)	1.08 (0.78, 1.49)
365-days	1.64 (1.16, 2.31)*	1.68 (1.18, 2.39)*	1.08 (0.77, 1.52)	1.07 (0.75, 1.51)
4-years	1.63 (1.19, 2.23)*	1.66 (1.21, 2.29)*	1.06 (0.77, 1.44)	1.05 (0.76, 1.44)
Note: CESD, Center for Epidemiological Studies–Depression; HADS-A, Hospital Anxiety and Depression Scale–anxiety subscale. ^***a***^Basic models adjust for age, sex, race/ethnicity, year, season, day of week, region and residence within a metropolitan statistical area (MSA). ^***b***^Multivariable models adjusted for age, sex, race/ethnicity, year, season, day of week, region, residence within a MSA, education attainment and family income of the participants, and median household income, percentage of population below poverty level in the census tract of residence. *p < 0.05.				

## Discussion

In our nationally representative sample of U.S. older adults, we observed statistically significantly positive associations with moderate-to-severe anxiety symptoms for all PM_2.5_ exposure windows (e.g., OR = 1.55; 95% CI: 1.31, 1.85 for PM_2.5_ averaged over 180 days). We also found increased odds of moderate-to-severe depressive symptoms associated with a 5 μg/m^3^ increment in PM_2.5_ exposure, with the largest increase associated with PM_2.5_ averaged over 30 days (OR = 1.20; 95% CI: 1.08, 1.33). Patterns of associations remain in multivariable models adjusting for SES. The observed associations were enhanced among individuals who were of low SES or had history of certain health-related conditions.

We found that reported depressive and anxiety symptoms at Wave 1 were only weakly correlated with corresponding symptoms at Wave 2. This supports our assumption that each mental health condition is reversible (Bedrosian et al. 2013; NIH/NIMH 2017), and is consistent with the CES-D and HADS-A questionnaires that evaluate current rather than chronic depression and anxiety symptoms. However, when depression and anxiety were considered as chronic conditions using logistic regression, PM_2.5_ was significantly associated with incident moderate-to-severe depressive symptoms in Wave 2 for all exposure windows examined, with higher effect estimates as compared to when depressive symptoms were assumed to be reversible disorders. In contrast, PM_2.5_ exposures were significantly associated with incident moderate-to-severe anxiety symptoms only for shorter exposure windows. These findings, which should be interpreted with caution, suggest that shorter-term PM_2.5_ exposures might be more biologically relevant to incident anxiety symptoms. However, associations of chronic PM_2.5_ exposure with anxiety should not be ruled out, as the decreased between-subject variability in long-term PM_2.5_ exposures leads to wider confidence intervals.

This study provides among the first evidence of positive associations between ambient PM_2.5_ exposures and adverse mental health symptoms, and is the first study to report increased odds of moderate-to-severe depressive symptoms associated with PM_2.5_ exposure. To date, only three epidemiologic studies have examined the association of long-term exposure to PM with mental health risk (Mehta et al. 2015; Power et al. 2015; Wang et al. 2014). Our observed positive and significant association between PM_2.5_ and moderate-to-severe anxiety symptoms is consistent with findings from Power et al. (2015) who also reported positive association of PM_2.5_ with phobic anxiety among a cohort of U.S. nurses, thus lending support for our findings. Yet, our findings of increased odds of depressive symptoms associated with PM_2.5_ differs from those of a Boston study that found a significant negative association (Wang et al. 2014). The conflicting findings may be attributed to our study’s larger sample size, use of participant-specific exposure measures, greater geographical coverage, and longer exposure windows examined. Other studies have reported positive and statistically significant association between short-term PM exposure (e.g., 1–3 lagged day) and suicidal risk (Bakian et al. 2015; Kim et al. 2010; Szyszkowicz et al. 2010), which may lend support to our findings of increased incident anxiety symptoms with semi-acute PM_2.5_ exposure windows.

While the biological pathways through which PM_2.5_ exposures influence mental health remain unknown, PM_2.5_ exposures may harm mental health through increased neuroinflammation, oxidative stress, cerebrovascular damage and neurodegeneration (Block and Calderón-Garcidueñas 2009; MohanKumar et al. 2008), as evidenced by findings from animal studies that show associations between PM and elevated hippocampal pro-inflammatory cytokine expression (Campbell et al. 2005; Fonken et al. 2011), upregulated expression of innate immunity and oxidative stress pathways (Sama et al. 2007), robust inflammatory and stress protein brain responses (Calderón-Garcidueñas et al. 2003), neuropathological damage in the brains of Apo E-deficient mice (Veronesi et al. 2005), and depression-like responses in mice (Fonken et al. 2011). PM_2.5_ pollution may also harm mental health by increasing markers of glucocorticoid activity and levels of the stress hormone cortisol (Thomson et al. 2013; Tomei et al. 2003) or through aggravating major respiratory or cardiac medical conditions (Power et al. 2015; Wang et al. 2014). Cardiopulmonary diseases positively associated with PM, such as asthma and heart failure, are also associated with increased prevalence of depression and anxiety disorders (Aben et al. 2003; Aström 1996; Cully et al. 2009; Dossa et al. 2011; Maurer et al. 2008; Scott et al. 2007), possibly mediated through biological (e.g., chronic inflammation) and behavioral (e.g., fear, social isolation) mechanisms (Hsu et al. 2014; Loubinoux et al. 2012; Yohannes and Alexopoulos 2014). Our findings of effect modification of the PM-mental health associations by individuals who had stroke, heart failure, or hypertension provide support for the importance of PM-mediated aggravation of cardiopulmonary conditions and our findings of PM_2.5_-mediated impacts on adverse mental health symptoms. In addition, our evidence of effect modification by SES suggests that PM exposure may have stronger impacts on depression symptoms among individuals with lower SES. We found that more participants living in neighborhoods with a greater percentage of the population with income below poverty level also had higher ambient PM_2.5_ pollution level near their residences; previous studies reported greater psychological stress and adverse mental health among people living in census tracts with lower SES and higher unemployment and poverty proportions (Bell and Ebisu 2012; Schwartz et al. 2011). Thus, a combination of greater pollution exposure and susceptibility may best explain how SES modified the association between PM exposure and depression symptoms.

Our study has several limitations. First, CESD and HADS-A are not clinical diagnostic instruments, nor are they designed to assess chronic mental disorder. Also, dichotomizing the continuous scores will likely reduce statistical power (Greenland 1995). However, these questionnaires are widely used screening tools for current level of depressive and anxiety symptom severity in the somatic, psychiatric and general population settings (Bjelland et al. 2002; Radloff 1977); and they provide cutoff scores for probable cases that are of clinical relevance, with high sensitivity, specificity and internal consistency (Dozeman et al. 2011). Second, residual confounding or confounding by unmeasured covariates and/or pollution (e.g., traffic noise pollution) is possible. Nonetheless, adjustment for several known confounding variables, including those related to SES and behaviors, did not eliminate the observed most significant associations and positive trends of PM_2.5_ with our mental health measures. Third, we assessed PM_2.5_ exposures using individual-specific exposures based on the nearest grid point values to the residential addresses, with the average distance of 2.23 km. While more precise than nearest monitor values, they do not account for time spent indoors or personal behaviors and thus are imperfect proxies of personal PM_2.5_ exposures and thus contribute to exposure misclassification. Last, findings from the current study may not be generalizable to younger age groups.

The nationally representative sample of older, community-dwelling Americans was a major strength of our study, since previous research of PM and mental health used convenience samples. We evaluated two affective measures to provide a comprehensive picture of air pollution’s impact on mental health, rather than one mental health measure as in existing studies. Our study was well-powered to detect meaningful associations and adjusted for confounding from individual- and census-level SES measures. Moreover, we showed effect modification of the PM_2.5_-mental health associations by participant characteristics, providing insight into susceptibility. We also considered multiple PM_2.5_ exposure windows; consistent with a previous study (Power et al. 2015), we found that intermediate-term PM_2.5_ exposure (e.g., 30 to 180 days) may be the most biological relevant exposure period to adverse mental symptoms, compared to longer exposure windows. Lastly, our findings were robust to multiple sensitivity analyses.

## Conclusions

We reported evidence of positive association between PM_2.5_ and moderate-to-severe depressive and anxiety symptoms among a representative sample of U.S. older adults. Our findings suggest that people with low SES or with a history of underlying health conditions may be more susceptible to increased odds of mental disorders after PM exposure.

## Supplemental Material

(637 KB) PDF
