# Supplementary material for: Association of Ambient Air Pollution with Depressive and Anxiety Symptoms in Older Adults: Results from the NSHAP Study
Source: Environ Health Perspect. 2016 Aug 12;125(3):342–8. doi: 10.1289/EHP494 (PMC5332196; doi:10.1289/EHP494)
Supplement: (637 KB) PDF [file EHP494.s001.acco.pdf]

Environ Health Perspect
DOI: 10.1289/EHP494

Note to readers with disabilities: *EHP* strives to ensure that all journal content is accessible to all readers. However, some figures and Supplemental Material published in *EHP* articles may not conform to 508 standards due to the complexity of the information being presented. If you need assistance accessing journal content, please contact ehp508@niehs.nih.gov. Our staff will work with you to assess and meet your accessibility needs within 3 working days.

Supplemental Material

Association of Ambient Air Pollution with Depressive and Anxiety Symptoms in Older Adults: Results from the NSHAP Study

Vivian C. Pun, Justin Manjourides, and Helen Suh

Table of Contents

Figure S1 Flowchart of participants through the NSHAP study
Figure S2 ORs (95% CIs) for mental illness per 5 μg/m^3 increment in $PM_{2.5}$ levels over extended range of exposure moving averages in multivariable model.
Table S1 Assessment of depression, anxiety, and perceived stress measures in the NSHAP study
Table S2 Description of covariates in the NSHAP study
Table S3 Interquartile range of $PM_{2.5}$ moving averages
Table S4 Characteristics of NSHAP study participants by waves and $PM_{2.5}$ pollution categories
Table S5 ORs (95% CIs) for mental disorders per 5 μg/m^3 increment in $PM_{2.5}$ levels over preceding 30 days moving average – evaluating potential confounding
Table S6 ORs (95% CI) for mental illness per 5 μg/m^3 increment in $PM_{2.5}$ levels over various moving averages – comparison of imputation methods using multivariable models
Table S7 ORs (95% CIs) for mental illness per 5 μg/m^3 increment in $PM_{2.5}$ levels over various preceding days moving averages – Restricting to only individuals who participated in both waves
Table S8 Percent change (95% CIs) in scores of mental health indicators per 5 μg/m^3 increment in $PM_{2.5}$ levels over preceding days moving averages
Table S9 Sensitivity analyses of the ORs (95% CIs) for mental disorders per 5 μg/m^3 increment in $PM_{2.5}$ levels over various preceding days moving averages in multivariable models
Table S10 Additional effect modification analysis of the association of mental illness with 5 μg/m^3 increment in $PM_{2.5}$ levels over preceding 30 days moving average in multivariable models with interaction terms for the potential modifier

Figure S1. Flowchart of participants through the NSHAP study[1].

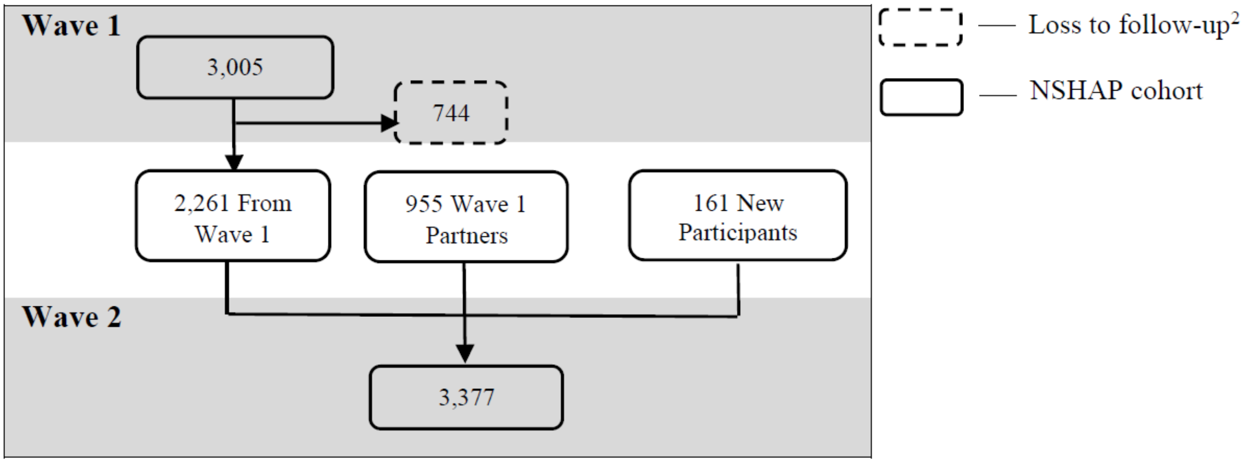

[1] The final analysis included 2,911 participants from Wave 1 and 3,288 participants from Wave 2, who had non-missing data for all covariates; [2] There were 744 Wave 1 Respondents who were either deceased or whose health was too poor to participate in Wave 2.

Table S1. Assessment of depression and anxiety measures in the NSHAP study.

	Rarely or none of the time	Some of the time	Occasionally	Most of the time
**Center for Epidemiological Studies – Depression (CESD-11)**				
(1) During the past week I did not feel like eating; my appetite was poor.	0	1	2	3
(2) During the past week I felt depressed.	0	1	2	3
(3) During the past week I felt that everything I did was an effort.	0	1	2	3
(4) During the past week my sleep was restless.	0	1	2	3
(5) During the past week I was happy.	3	2	1	0
(6) During the past week I felt lonely.	0	1	2	3
(7) During the past week people were unfriendly.	0	1	2	3
(8) During the past week I enjoyed life.	3	2	1	0
(9) During the past week I felt sad.	0	1	2	3
(10) During the past week I felt that people disliked me.	0	1	2	3
(11) During the past week I could not get "going."	0	1	2	3
**Hospital Anxiety and Depression Scale – Anxiety subscale (HADS-A)**				
(1) During the past week I felt tense or "wound up."	0	1	2	3
(2) During the past week I got a frightened feeling as if something awful was about to happen.	0	1	2	3
(3) During the past week worrying thoughts went through my mind.	0	1	2	3
(4) During the past week I could sit at ease and feel relaxed.	3	2	1	0
(5) During the past week I got a frightened feeling like butterflies in my stomach.	0	1	2	3
(6) During the past week I felt restless as if I had to be on the move.	0	1	2	3
(7) During the past week I had a sudden feeling of panic.	0	1	2	3

Table S2. Description of covariates in the NSHAP study.

Variable	Description	Assessment
Body mass index	Calculated using measured height and weight (kg)	Kg/m^2
Current smoking status	Questionnaire: "Do you smoke cigarettes now, not including pipes, snug, chewing tobacco or any other forms of tobacco besides cigarettes)?"	Yes, No
Physical activity	Questionnaire: "How often do you participate in physical activity such as walking, dancing, gardening, physical exercise or sports?"	Never, less than 1 time per month, 1 - 3 times per month, 1 - 2 times per week, 3 or more times per week
Alcohol consumption	Questionnaire: "In the last three months, on the days you drink, about how many drinks do you have?"	Drinks per day
UCLA Loneliness scale	Summed scores from three questions indicating loneliness – "I lack of companionship," "I feel left out," and "I feel isolated."	Ranges 0 to 9; higher scores indicate feeling lonely most of the time.
Any history of the following health condition: diabetes, hypertension, stroke, heart failure, emphysema, chronic obstructive pulmonary disease (COPD) or asthma	Questionnaire: "Has a doctor ever told you that you have diabetes?" "Has a doctor ever told you that you have high blood pressure or hypertension?" "Has a doctor ever told you that you have stroke?" "Has a doctor ever told you that you have heart failure?" "Has a doctor ever told you that you have emphysema, chronic obstructive pulmonary disease (COPD) or asthma?"	Yes, No
Antidepressant medication	Current use reported by participants	Yes, No
Family income	Questionnaire: "Now, I'd like to ask you about the income of your household. Altogether, what would you say was approximately the income of your household in last year before taxes or deductions?"	$

Table S3. Interquartile range of $PM_{2.5}$ moving averages

$PM_{2.5}$ moving averages	IQR ($\mu g/m^3$)
7-days	6.1
30-days	5.6
180-days	4.3
365-days	3.9
4-years	3.7

Table S4. Characteristics of NSHAP study participants by waves and $PM_{2.5}$ pollution categories.

Variable	$PM_{2.5}$ pollution categories: Wave 1*			$PM_{2.5}$ pollution categories: Wave 2*		
	Low	Median	High	Low	Median	High
**No. of participants**	752	1,503	750	846	1,681	850
**Age** (year, mean ± SD[a])	69.4 ± 8.0	69.1 ± 7.8	69.6 ± 7.8	72.2 ± 8.3	72.2 ± 8.0	72.9 ± 8.0
**Male** (%)	51.6	48.0	46.0	48.5	45.2	43.3
**Race** (%)						
White	83.1	685	62.0	87.9	69.2	59.3
Black	3.5	19.7	25.3	1.7	18.1	23.7
Hispanic non-black	11.3	10.1	9.0	8.1	11.1	13.4
Other	2.1	1.7	3.8	2.4	1.6	3.7
**BMI**[b] (kg/m^2, mean ± SD)	28.8 ± 6.1	29.2 ± 6.1	29.2 ± 6.2	29.1 ± 6.1	29.3 ± 5.9	29.6 ± 6.4
Obesity (% ≥30 BMI)	32.5	35.5	36.9	35.0	37.2	37.3
**Alcohol consumption** (drinks/day, mean ± SD)	1.2 ± 1.5	1.0 ± 1.5	1.1 ± 1.7	1.0 ± 1.5	0.9 ± 1.4	1.0 ± 1.5
**Current smoking** (%)	14.1	14.7	15.8	11.7	13.0	15.5
**Physical activity** (%)						
3 or more times per week	69.1	59.4	57.9	45.2	39.9	38.3
1-2 times per week	12.5	15.9	17.4	15.3	16.1	14.6
1-3 times per month	5.5	6.5	6.8	8.8	8.5	9.0
Less than 1 time per month	5.2	6.6	7.3	10.3	9.0	9.0
Never	7.7	11.6	10.6	20.5	26.5	29.1
**Socioeconomic status**						
Individual-level						
Education attainment (%)						
College degree or greater	28.6	20.4	18.1	31.9	23.5	19.1
High school or vocational school	55.7	52.8	58.3	57.2	54.6	59.3
Less than high school	15.7	26.9	23.6	10.9	22.0	21.7
Family income ($ in thousands, mean ± SD)	56.4 ± 69.5	48.8 ± 50.3	51.1 ± 81.6	65.5 ± 74.9	58.2 ± 68.6	55.6 ± 83.2
% ≤$35,000	37.2	37.7	38.1	28.5	30.7	34.1
Census-level[d]						
Median household income ($ in thousands, mean ± SD)	55.3 ± 25.8	52.2 ± 25.5	51.1 ± 23.5	61.2 ± 28.6	56.2 ± 26.5	52.2 ± 26.8
Population with income below poverty level (%)	12.6	16.1	16.3	11.0	14.4	17.8

* <25th percentile as Low, 25th–75th percentile as Median, and >75th percentile as High; [a] SD refers to standard deviation; [b] BMI refers to body mass index; [c] COPD refers to chronic obstructive pulmonary disease; [d] Estimated for census tract of residence using data from the US Census Bureau (2000); [e] CESD stands for the Center for Epidemiological Studies – Depression; [f] HADS-A stands for the Hospital Anxiety and Depression Scale – anxiety subscale; [g] PSS stands for Perceived Stress Scale.

Table S4 (cont'). Characteristics of NSHAP study participants by waves and $PM_{2.5}$ pollution categories.

Variable	$PM_{2.5}$ pollution categories: Wave 1*			$PM_{2.5}$ pollution categories: Wave 2*		
	Low	Median	High	Low	Median	High
**Loneliness score** (mean ± SD)	3.9 ± 1.4	4.0 ± 1.4	4.2 ± 1.5	3.0 ± 2.2	3.0 ± 2.3	3.1 ± 2.3
**Diabetes (%)**	17.4	22.3	23.5	20.1	24.7	25.5
**Hypertension (%)**	51.7	58.2	61.1	58.0	62.6	63.4
**Stroke** (%)	8.1	9.0	9.6	9.5	9.1	9.8
**Heart failure** (%)	7.0	10.4	10.3	5.6	4.1	6.1
**Emphysema, COPD[c] or asthma** (%)	16.1	17.8	17.3	17.8	14.6	14.6
**Antidepressant use (%)**	12.4	12.1	13.4	15.3	15.0	15.3
**CESD-11 score[e]** (mean ± SD)	5.1 ± 4.9	5.7 ± 5.3	5.9 ± 5.4	4.7 ± 4.8	5.0 ± 4.8	5.5 ± 5.3
Number (%) ≥9	20.7	25.3	26.0	19.0	19.8	24.6
**HADS-A score[f]** (mean ± SD)	3.2 ± 3.2	3.6 ± 3.5	3.9 ± 3.7	4.4 ± 3.6	4.8 ± 3.6	4.9 ± 3.7
Number (%) ≥8	10.1	14.0	16.0	18.7	21.5	23.8
**$PM_{2.5}$ annual concentration** ($\mu g/m^3$, mean ± SD)	7.1 ± 1.9	11.2 ± 1.2	14.6 ± 1.0	5.7 ± 1.3	8.9 ± 0.9	11.5 ± 0.9

* <25th percentile as Low, 25th–75th percentile as Median, and >75th percentile as High; [a] SD refers to standard deviation; [b] BMI refers to body mass index; [c] COPD refers to chronic obstructive pulmonary disease; [d] Estimated for census tract of residence using data from the US Census Bureau (2000); [e] CESD stands for the Center for Epidemiological Studies – Depression; [f] HADS-A stands for the Hospital Anxiety and Depression Scale – anxiety subscale; [g] PSS stands for Perceived Stress Scale.

Figure S2. ORs (95% CIs) for mental illness per 5 μg/m^3 increment in $PM_{2.5}$ levels over extended range of exposure moving averages in multivariable models[1].

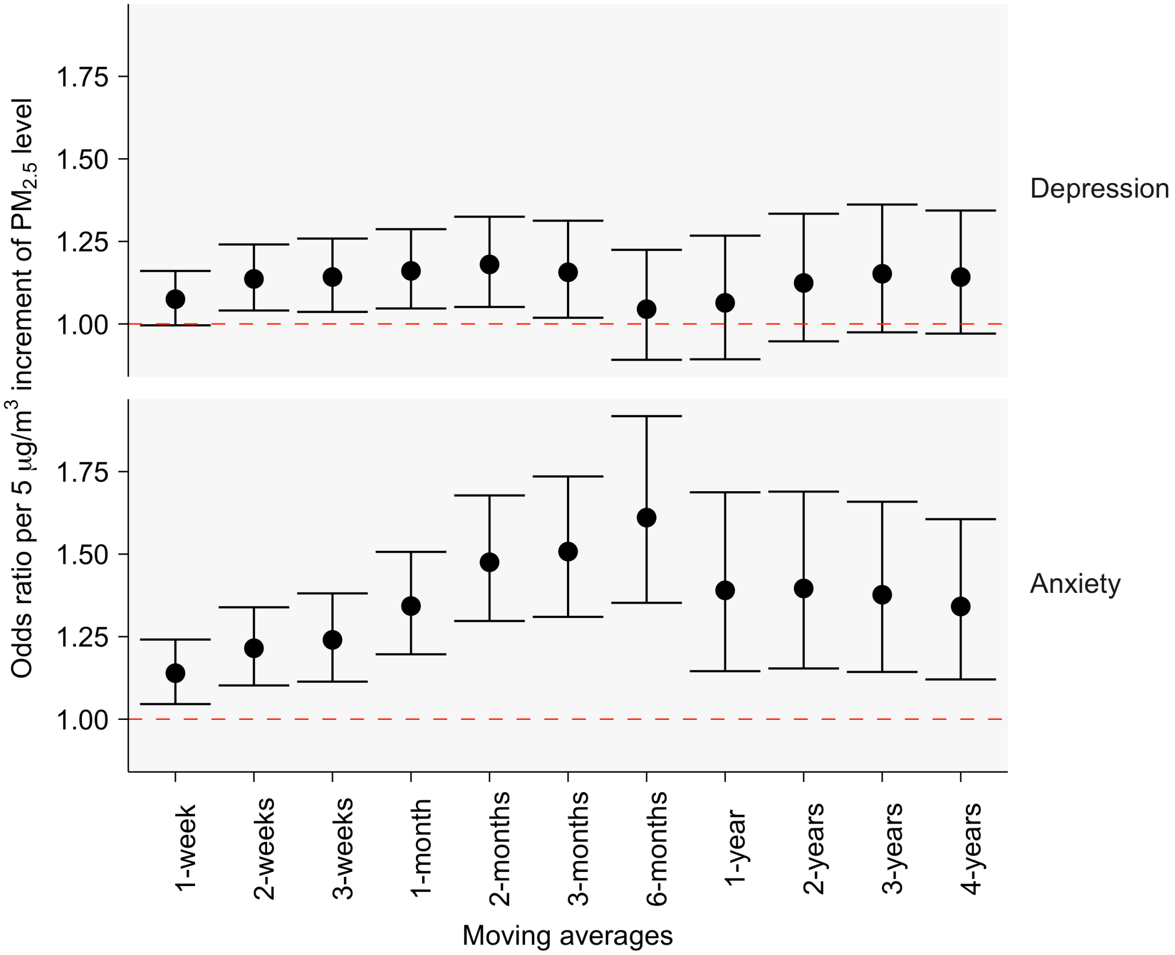

[1] Multivariable models adjusted for age, gender, race/ethnicity, year, season, day of week, region and residence within a MSA, education attainment and family income of the participants, and median household income, percentage of population below poverty level in the census tract of residence.

Table S5. ORs (95% CIs) for mental disorders per 5 μg/m^3 increment in $PM_{2.5}$ levels over preceding 30 days moving average – evaluating potential confounding.

Model covariates	Depression: CESD-11 ≥ 9 vs < 9	Anxiety: HADS-A ≥ 8 vs < 8
Basic[1]	1.199 (1.083, 1.328)*	1.343 (1.196, 1.507)*
BMI (≥30 vs. <30)	1.198 (1.081, 1.326)*	1.342 (1.197, 1.504)*
Alcohol consumption	1.184 (1.069, 1.313)*	1.319 (1.176, 1.480)*
Smoking status	1.196 (1.081, 1.325)*	1.338 (1.194, 1.500)*
Physical activity	1.188 (1.081, 1.316)*	1.339 (1.195, 1.500)*
Education attainment	1.178 (1.064, 1.304)*	1.326 (1.183, 1.486)*
Family income (≤35,000)	1.199 (1.083, 1.328)*	1.341 (1.196, 1.502)*
Median household income	1.177 (1.061, 1.305)*	1.348 (1.202, 1.513)*
Population with income below poverty level	1.169 (1.055, 1.297)*	1.345 (1.198, 1.509)*
UCLA Loneliness scale	1.153 (1.025, 1.296)*	1.308 (1.159, 1.475)*
Diabetes	1.191 (1.075, 1.319)*	1.336 (1.192, 1.497)*
Hypertension	1.199 (1.083, 1.328)*	1.337 (1.193, 1.498)*
Stroke	1.200 (1.083, 1.328)*	1.337 (1.194, 1.499)*
Heart failure	1.197 (1.080, 1.326)*	1.330 (1.187, 1.491)*
Emphysema, COPD or asthma	1.199 (1.082, 1.327)*	1.333 (1.189, 1.494)*
Current use of antidepressants	1.192 (1.076, 1.320)*	1.338 (1.192, 1.502)*

[1] Basic models adjust for age, gender, race/ethnicity, year, season, day of week, region and residence within a MSA.
* P<0.05.

Table S6. ORs (95% CI) for mental illness per 5 μg/m^3 increment in $PM_{2.5}$ levels over various moving averages – comparison of imputation methods using multivariable models[1]

$PM_{2.5}$ moving averages	Depression: CESD-11 ≥ 9 vs < 9		Anxiety: HADS-A ≥ 8 vs < 8	
	Simple mean substitution	Multiple imputation[2]	Simple mean substitution	Multiple imputation[2]
7-days	1.08 (1.00, 1.16)**	1.07 (1.00, 1.16)**	1.14 (1.05, 1.24)*	1.14 (1.04, 1.24)*
30-days	1.16 (1.05, 1.29)*	1.16 (1.05, 1.29)*	1.31 (1.20, 1.51)*	1.34 (1.19, 1.50)*
180-days	1.04 (0.89, 1.22)	1.04 (0.89, 1.22)	1.61 (1.35, 1.92)*	1.60 (1.34, 1.91)*
365-days	1.06 (0.89, 1.27)	1.06 (0.89, 1.26)	1.39 (1.15, 1.69)*	1.38 (1.14, 1.68)*
4-years	1.14 (0.97, 1.34)	1.14 (0.97, 1.34)	1.34 (1.12, 1.61)*	1.33 (1.11, 1.60)*

[1] Multivariable models adjusted for age, gender, race/ethnicity, year, season, day of week, region, residence within a MSA, education attainment and family income of the participants, and median household income, percentage of population below poverty level in the census tract of residence.
[2] Missing data was filled in using multiple imputation technique following a Markov Chain Monte Carlo distribution.
* P<0.05; ** P<0.10.

Table S7. ORs (95% CIs) for mental illness per 5 μg/m^3 increment in $PM_{2.5}$ levels over various preceding days moving averages – Restricting to only individuals who participated in both waves.

$PM_{2.5}$ moving averages	Depression: CESD-11 ≥ 9 vs < 9		Anxiety: HADS-A ≥ 8 vs < 8	
	Basic[1]	Multivariable adjusted[2]	Basic[1]	Multivariable adjusted[2]
7-days	1.12 (1.03, 1.23)*	1.11 (1.02, 1.22)*	1.17 (1.05, 1.29)*	1.14 (1.03, 1.27)*
30-days	1.28 (1.13, 1.45)*	1.24 (1.09, 1.40)*	1.29 (1.12, 1.49)*	1.26 (1.10, 1.46)*
180-days	1.10 (0.91, 1.34)	1.02 (0.83, 1.24)	1.57 (1.27, 1.95)*	1.54 (1.24, 1.92)*
365-days	1.19 (0.96, 1.47)	1.10 (0.89, 1.37)	1.36 (1.07, 1.73)*	1.34 (1.05, 1.70)*
4-years	1.31(1.07, 1.60)*	1.24 (1.01, 1.51)	1.32 (1.06, 1.64)*	1.31 (1.05, 1.63)*

[1] Basic models adjust for age, gender, race/ethnicity, year, season, day of week, region and residence within a MSA.
[2] Multivariable models adjusted for age, gender, race/ethnicity, year, season, day of week, region, residence within a MSA, education attainment and family income of the participants, and median household income, percentage of population below poverty level in the census tract of residence.
* P<0.05; ** P<0.10.

Table S8. Percent change (95% CIs) in scores of mental health indicators per 5 μg/m^3 increment in $PM_{2.5}$ levels over preceding days moving averages.

$PM_{2.5}$ moving averages	Depression: CESD-11 (range: 0-33)		Anxiety: HADS-A (range: 0-21)	
	Basic[1]	Multivariable adjusted[2]	Basic[1]	Multivariable adjusted[2]
7-days	2.72 (0.18, 5.33)*	2.51 (-0.03, 5.11)**	1.22 (-1.19, 3.69)	1.26 (-1.17, 3.74)
30-days	4.16 (0.81, 7.62)*	3.62 (0.29, 7.07)*	3.34 (0.17, 6.61)*	3.23 (0.04, 6.52)*
180-days	2.15 (-2.67, 7.20)	1.77 (-3.04, 6.81)	7.51 (2.83, 12.40)*	7.79 (3.05, 12.74)*
365-days	2.22 (-3.08, 7.80)	2.39 (-2.91, 7.98)	4.53 (-0.46, 9.78)**	4.97 (-0.08, 10.29)**
4-years	3.64 (-1.37, 8.89)	3.83 (-1.18, 9.10)	3.98 (-0.66, 8.83)**	4.29 (-0.40, 9.19)**

[1] Basic models adjust for age, gender, race/ethnicity, year, season, day of week, region and residence within a MSA.
[2] Multivariable models adjusted for age, gender, race/ethnicity, year, season, day of week, region, residence within a MSA, education attainment and family income of the participants, and median household income, percentage of population below poverty level in the census tract of residence.
* P<0.05; ** P<0.10.

Table S9. Sensitivity analyses of the ORs (95% CIs) for mental disorders per 5 μg/m^3 increment in $PM_{2.5}$ levels over various preceding days moving averages in multivariable models[1].

	Depression	Anxiety
**Primary Analyses**		
7-days	1.08 (1.00, 1.16)**	1.14 (1.05, 1.24)*
30-days	1.16 (1.05, 1.29)*	1.31 (1.20, 1.51)*
180-days	1.04 (0.89, 1.22)	1.61 (1.35, 1.92)*
365-days	1.06 (0.89, 1.27)	1.39 (1.15, 1.69)*
4-years	1.14 (0.90, 1.34)	1.34 (1.12, 1.61)*
**Sensitivity – Restriction to participants who did not take antidepressant medication**		
7-days	1.03 (0.94, 1.12)	1.04 (0.94, 1.15)
30-days	1.06 (0.95, 1.19)	1.22 (1.07, 1.39)*
180-days	0.90 (0.76, 1.08)	1.49 (1.22, 1.82)*
365-days	0.90 (0.74, 1.10)	1.26 (1.01, 1.57)*
4-years	0.98 (0.82, 1.17)	1.22 (1.00, 1.51)**
**Sensitivity – Restriction to ambient monitor stations within 60 kilometer**		
7-days	1.01 (0.95, 1.08)	1.13 (1.04, 1.21)*
30-days	1.07 (0.97, 1.17)	1.13 (1.01, 1.26)*
180-days	0.97 (0.83, 1.12)	1.20 (1.02, 1.42)*
365-days	0.95 (0.79, 1.14)	1.00 (0.82, 1.23)
4-years	0.98 (0.82, 1.16)	0.96 (0.79, 1.16)
**Sensitivity – Restriction to those living in a MSA**[2]		
7-days	1.04 (0.96, 1.13)	1.09 (1.00, 1.20)**
30-days	1.11 (1.00, 1.24)*	1.35 (1.19, 1.52)*
180-days	0.99 (0.84, 1.17)	1.65 (1.37, 2.00)*
365-days	0.98 (0.82, 1.18)	1.46 (1.18, 1.80)*
4-years	1.07 (0.90, 1.27)	1.43 (1.17, 1.74)*
**Sensitivity – Restriction to non-movers**		
7-days	1.05 (0.96, 1.15)	1.17 (1.06, 1.29)*
30-days	1.11 (0.99, 1.26)	1.39 (1.21, 1.59)*
180-days	0.99 (0.82, 1.20)	1.63 (1.32, 2.00)*
365-days	1.01 (0.82, 1.25)	1.33 (1.05, 1.67)*
4-years	1.03 (0.84, 1.25)	1.36 (1.09, 1.69)*

[1] Multivariable models adjusted for age, gender, race/ethnicity, year, season, day of week, region, residence within a MSA, education attainment and family income of the participants, and median household income, percentage of population below poverty level in the census tract of residence.
[2] Multivariable models adjusted for all covariates but residence within a MSA.
* P<0.05; ** P<0.10.

Table S10. Additional effect modification analysis of the association of mental illness with 5 μg/m^3 increment in $PM_{2.5}$ levels over preceding 30 days moving average in multivariable models[1].

Effect modifier	Depression: CESD-11 ≥ 9 vs < 9		Anxiety: HADS-A ≥ 8 vs < 8	
**Age**	OR (95% CI)	$p_{interact}$	OR (95% CI)	$p_{interact}$
≤70	1.16 (1.02, 1.31)		1.38 (1.20, 1.59)	
>70	1.06 (0.93, 1.22)	0.283	1.36 (1.17, 1.59)	0.892
**Race**				
White	1.06 (0.95, 1.20)		1.46 (1.28, 1.65)	
Black	1.42 (1.15, 1.76)	0.009	1.17 (0.90, 1.54)	0.122
Hispanic non-black	1.13 (0.84, 1.52)	0.703	1.30 (0.92, 1.84)	0.528
Other	0.85 (0.50, 1.42)	0.393	0.45 (0.23, 0.89)	0.001
**Region**				
West	1.09 (0.89, 1.32)		1.26 (1.01, 1.57)	
Midwest	1.17 (0.97, 1.42)	0.563	1.35 (1.10, 1.67)	0.621
South	1.21 (1.04, 1.40)	0.367	1.36 (1.15, 1.61)	0.540
Northeast	0.93 (0.76, 1.13)	0.241	1.53 (1.25, 1.87)	0.173
**Season**				
Spring	0.45 (0.28, 0.73)		0.96 (0.59, 1.55)	
Summer	1.16 (1.00, 1.33)	<0.001	1.33 (1.13, 1.57)	0.200
Fall	1.15 (0.98, 1.34)	<0.001	1.43 (1.21, 1.69)	0.111
Winter	1.11 (0.85, 1.46)	0.001	1.50 (1.11, 2.02)	0.121

[1] Multivariable models adjusted for age, gender, race/ethnicity, year, season, day of week, region, residence within a MSA, education attainment and family income of the participants, and median household income, percentage of population below poverty level in the census tract of residence.
